# Low‐energy amplitude‐modulated electromagnetic field exposure: Feasibility study in patients with hepatocellular carcinoma

**DOI:** 10.1002/cam4.5944

**Published:** 2023-05-15

**Authors:** Fernanda Capareli, Frederico Costa, Jack A. Tuszynski, Micelange C. Sousa, Yone de C. Setogute, Pablo D. Lima, Luciana Carvalho, Elizabeth Santos, Brenda P. Gumz, Jorge Sabbaga, Tiago B. de Castria, Denis L. Jardim, Daniela Freitas, Natally Horvat, Regis O. F. Bezerra, Leonardo Testagrossa, Tiago Costa, Tatiana Zanesco, Antonio F. Iemma, Ghassan K. Abou‐Alfa

**Affiliations:** ^1^ Oncology Department Hospital Sírio‐Libanês São Paulo Brazil; ^2^ Autem Medical LLC Hanover New Hampshire USA; ^3^ Division of Experimental Oncology, Department of Oncology Cross Cancer Institute, University of Alberta Edmonton Alberta Canada; ^4^ Oncology Department A. C. Camargo Cancer Center São Paulo Brazil; ^5^ Memorial Sloan Kettering Cancer Center New York New York USA; ^6^ Radiology Department Hospital Sírio‐Libanês São Paulo Brazil; ^7^ Pathology Department Hospital Sírio‐Libanês São Paulo Brazil; ^8^ Santa Casa de São Paulo School of Medical Sciences São Paulo Brazil; ^9^ Institute of Mathematics and Statistics, University of São Paulo São Paulo Brazil; ^10^ Weill Medical College at Cornell University New York New York USA

**Keywords:** EMF, hemodynamics, hepatocellular carcinoma, low‐frequency electromagnetic fields, safety

## Abstract

**Background:**

Patients with advanced hepatocellular carcinoma (HCC) and poor liver function lack effective systemic therapies. Low‐energy electromagnetic fields (EMFs) can influence cell biological processes via non‐thermal effects and may represent a new treatment option.

**Methods:**

This single‐site feasibility trial enrolled patients with advanced HCC, Child‐Pugh A and B, Eastern Cooperative Oncology Group 0–2. Patients underwent 90‐min amplitude‐modulated EMF exposure procedures every 2–4 weeks, using the AutEMdev (Autem Therapeutics). Patients could also receive standard care. The primary endpoints were safety and the identification of hemodynamic variability patterns. Exploratory endpoints included health‐related quality of life (HRQoL), overall survival (OS). and objective response rate (ORR) using RECIST v1.1.

**Results:**

Sixty‐six patients with advanced HCC received 539 AutEMdev procedures (median follow‐up, 30 months). No serious adverse events occurred during procedures. Self‐limiting grade 1 somnolence occurred in 78.7% of patients. Hemodynamic variability during EMF exposure was associated with specific amplitude‐modulation frequencies. HRQoL was maintained or improved among patients remaining on treatment. Median OS was 11.3 months (95% confidence interval [CI]: 6.0, 16.6) overall (16.0 months [95% CI: 4.4, 27.6] and 12.0 months [6.4, 17.6] for combination therapy and monotherapy, respectively). ORR was 24.3% (32% and 17% for combination therapy and monotherapy, respectively).

**Conclusion:**

AutEMdev EMF exposure has an excellent safety profile in patients with advanced HCC. Hemodynamic alterations at personalized frequencies may represent a surrogate of anti‐tumor efficacy. NCT01686412.

## 1 BACKGROUND

Hepatocellular carcinoma (HCC) is a major global health issue that primarily affects patients with cirrhosis.^1^ Most patients with unresectable disease have a poor prognosis,^2^, ^3^ and many with advanced HCC cannot tolerate systemic therapy.^3^, ^4^ Poor liver function in patients with advanced HCC arises from tumor‐associated loss of parenchyma and damage by anti‐cancer therapies.^5^

**FIGURE 1:**
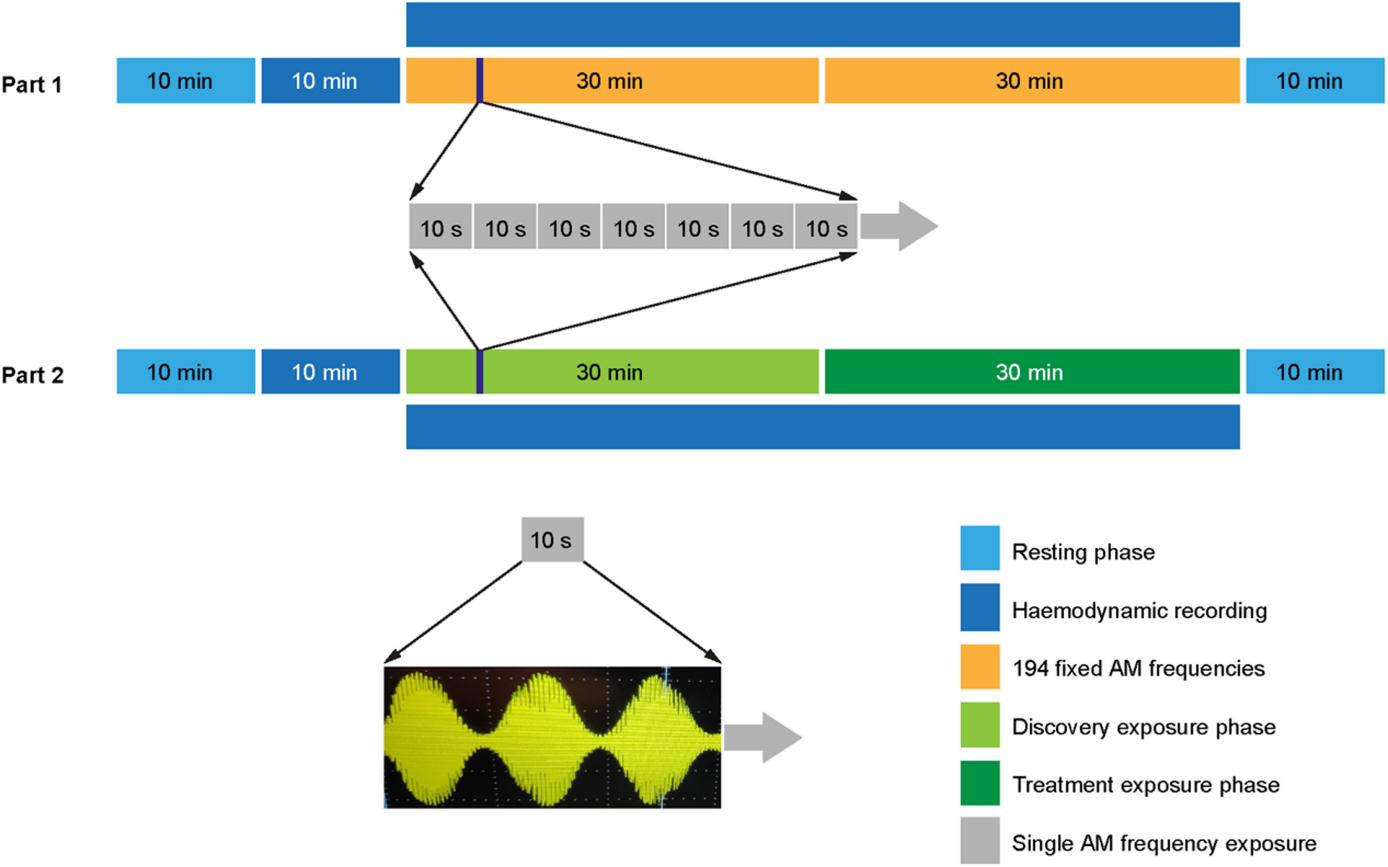
Schematic of EMF exposure procedure using the AutEMdev in study parts 1 and 2. The bottom panel depicts amplitude modulation of the low‐energy 27.12 MHz carrier wave (rapid oscillation; short wavelength) to produce a therapeutic envelope wave with a lower frequency (slow oscillation, long wavelength), which can be any specific frequency in the range from 10 Hz to 20 kHz. Abbreviations: AM indicates amplitude‐modulated; AutEMdev, Autem electromagnetic device; EMF, electromagnetic field.

Low‐energy radio frequency electromagnetic fields (EMFs) penetrate cells and can influence multiple cell biological processes via non‐thermal effects.^6^ Low‐energy EMFs may offer an alternative treatment for advanced HCC, in combination with conventional therapies or as a monotherapy.^7^, ^8^, ^9^ Modalities for EMF treatment in patients with cancer include the Autem electromagnetic device (AutEMdev) and tumor‐treating fields (TTFields). The AutEMdev technology involves intrabuccal delivery of EMFs to the whole body using a 27.12 MHz carrier frequency with amplitude modulation at specific frequencies in the range 10 Hz–20 kHz.^8^ The device power of 100 mW is far below than that of a mobile telephone.^10^ Amplitude modulation allows generation of different envelope waves with any frequency substantially lower than that of the carrier wave, as shown in Figure 1 and described in detail in a recent review.^10^ This modality had a good safety profile with evidence of anti‐tumor effects in a phase 1/2 trial in patients with advanced HCC^7^ and in a mouse xenograft model of HCC.^9^ TTFields uses low‐energy alternating electric fields of intermediate frequency (∼100–500 kHz).^11^ The US Food and Drug Administration approved TTFields as a monotherapy in patients with recurrent glioblastoma and mesothelioma, based on randomized phase 3 clinical trials.^12^, ^13^, ^14^, ^15^, ^16^

Evidence indicates that cancer cells may be more susceptible to perturbation by EMFs than normal cells. Ions and molecules with a high electrical dipole moment are susceptible to frequency‐dependent interactions with EMFs.^17^, ^18^, ^19^ EMFs, therefore exert substantial dielectrophoretic forces on microtubules, which may disrupt cell division.^11^, ^20^ Transmembrane potentials drop from −90 to −20 mV or lower in cancer cells compared with normal cells, presumably because of alterations in ion channel dynamics.^21^, ^22^ Mitochondrial pH and potassium concentrations also change compared with normal cells.^23^, ^24^ These alterations may underlie the potentially beneficial effects of EMF exposure on cancer progression.^7^, ^9^, ^25^, ^26^

Low‐energy EMFs can also cause alterations in hemodynamic regulation. Although the underlying physiology is unclear, this may offer a route toward identification of potentially beneficial anti‐cancer effects. Heart rate variability and other beat‐to‐beat parameters can be analyzed using non‐linear computing methods as indicators of alterations in hemodynamic regulation during EMF exposure.^27^ We hypothesize that amplitude‐modulation frequencies that alter the behavior of electrically excitable cells may also disrupt the proliferation of cancer cells. This predicts a non‐random distribution of hemodynamically active frequencies. A hemodynamic surrogate measure may allow identification of personalized anti‐cancer amplitude‐modulation frequencies.^28^, ^29^, ^30^

We conducted an exploratory study of the AutEMdev in patients with advanced HCC and healthy controls. The study aimed to assess safety and feasibility, and to explore hemodynamic variability parameters as a means of personalizing EMF exposure.

## 2 METHODS

This was a prospective, open‐label study of the safety and feasibility of intrabuccal EMF exposure using the AutEMdev, with exploratory assessment of hemodynamic regulation alterations. The study had two sequential parts, both conducted in patients with HCC and healthy volunteers (Figure S1). Amplitude‐modulation frequencies were fixed in part 1 and variable in part 2, based on personalized frequencies associated with hemodynamic alterations (Figure 1). The study also included a historical control cohort.

### 2.1 Study population

Eligible patients were aged at least 18 years and had advanced HCC, defined as unresectable locally advanced or metastatic HCC, confirmed by histology or clinical presentation according to the American Association for the Study of Liver Diseases criteria.^31^ Child–Pugh A or B liver cirrhosis, Barcelona Clinic Liver Cancer stage B or C, and Eastern Cooperative Oncology Group performance status score of 0–2 were required.^32^, ^33^, ^34^, ^35^ There were no restrictions on disease progression status, hematological function, organ function, or current or previous therapy (including failure of all available systemic treatments). Eligible healthy volunteers were aged at least 18 years with no history of cancer. Key exclusion criteria for patients and healthy volunteers were cholangiocarcinoma, other active cancer, other life‐threatening medical conditions, implantable medical devices, and eligibility for loco‐regional therapy.

The historical control cohort was non‐interventional and retrospective, comprising patients with unresectable HCC identified from the Hospital Sírio‐Libanês Oncology Department electronic medical records using the keyword “liver cancer.” Patients had to have a diagnosis of advanced HCC after loco‐regional therapy, have been recommended for systemic therapy, or be receiving best supportive care.

### 2.2 Medical devices

Participants were exposed to low‐energy radio frequency EMFs using the AutEMdev (Autem Therapeutics). The AutEMdev is an autonomous high‐precision EMF generator designed to execute and control systemic exposure to low‐energy radio frequency EMFs with a carrier frequency of 27.12 MHz and sinusoidal amplitude modulation at frequencies ranging from 10 Hz to 20 kHz. EMF exposures via AutEMdev are within the safety levels published by the International Commission on Non‐Ionizing Radiation Protection.^36^ Hemodynamic regulation was assessed using a non‐invasive continuous beat‐to‐beat recording device (Task Force Monitor or CNAP500; CNSystems, Graz, Austria) synchronized with the AutEMdev.

### 2.3 Quality of life assessment

Patients with HCC were required to complete the European Organization for Research and Treatment of Cancer Quality of Life Questionnaire Core 30 (EORTC QLQ–C30)^37^ before each AutEMdev exposure procedure. Completion was defined as answering at least 90% of the questions.

### 2.4 Study procedures

Patients were assigned to at least one 90‐min outpatient AutEMdev exposure procedure. Healthy volunteers were assigned to one exposure procedure only. Patients were invited to repeat procedures every 2–4 weeks if the patient or physician perceived quality of life improvement or objective tumor response. Patients could continue until withdrawal of consent or deterioration in quality of life (10% decrease from baseline in EORTC QLQ–C30 global health status). Patients could receive AutEMdev as a monotherapy or in combination with any conventional treatment approved by the Brazilian health regulatory agency, at any time. All patients were allowed to maintain any supportive care.

**FIGURE 2:**
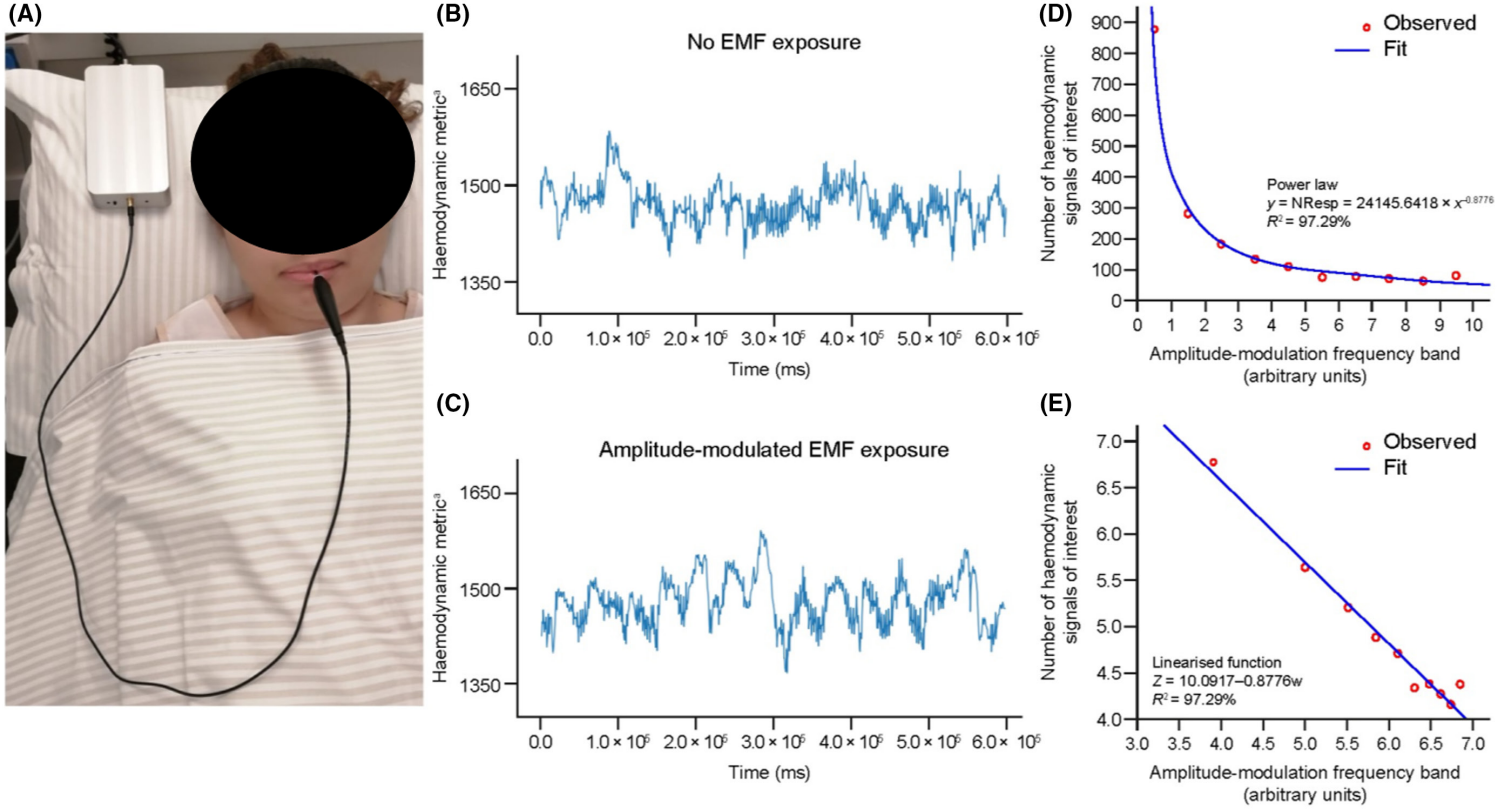
Hemodynamic variability alterations during EMF exposure with the AutEMdev. (A) Participant undergoing the procedure showing the AutEMdev and intrabuccal spoon‐shaped antenna. (B,C) Hemodynamic variability without and during exposure to amplitude‐modulated EMFs. (D) Power law distribution and (E) Linearized function for the number of hemodynamic signals of interest identified in 10 specific amplitude‐modulation frequency bands, following division of the time series into 100‐s intervals (data from study part 2). Abbreviations: AutEMdev indicates Autem electromagnetic device; EMF, electromagnetic field; NResp, number of responses (i.e., hemodynamic signals of interest).

Exposure procedures were conducted with continuous assistance from healthcare professionals. Participants were placed in the supine position wearing a comfortable outfit in a quiet, low‐light ambiance to allow hemodynamic parameters to stabilize. Pressure cuffs were positioned, and the spoon‐shaped AutEMdev antenna was placed over the patient's tongue (Figure 2A). After 10 min, beat‐to‐beat continuous hemodynamic recording started and continued for 10 min (without EMF exposure). EMF exposure and amplitude modulation then started in synchronization with hemodynamic recording/reading and continued for 60 min (Figure 1). Equipment was removed and participants got dressed during the final 10 min of the 90‐min procedure. Exposure was supervised throughout the procedure to allow detection of exposure‐related toxicity or device malfunction. The procedure was aborted if the participant showed signs of intolerability (e.g., inability to tolerate decubitus, extreme discomfort).

### 2.5 Study design

All participants underwent baseline hemodynamic recording without EMF exposure for 10 min, followed by hemodynamic recording with EMF exposure for 60 min (Figure 1).

In part 1, all participants received two 30‐min exposures to a pre‐programmed fixed range and sequence of 194 separate modulation frequencies via the AutEMdev (Figure 1). Modulation frequencies were applied individually in sequence from lowest (10 kHz) to highest (20 kHz) for 10 s each during each 30‐min exposure.

In part 2, all participants underwent a 30‐min discovery phase followed by a 30‐min individualized phase (Figure 1). In the discovery phase, participants were exposed to a pre‐programmed fixed range and sequence of modulation frequencies (different from part 1) via the AutEMdev. Modulation frequencies were applied individually in sequence from lowest to highest for 10 s each. The AutEMdev then automatically used data filtering and post‐processing to construct a new personalized range and sequence of modulation frequencies, by identifying and prioritizing frequencies associated with hemodynamic signals of interest. Each participant was then exposed sequentially to their unique personalized treatment frequencies (Figure 1).

### 2.6 Endpoints

The predefined primary endpoints were safety and the identification of hemodynamic variability induced by the EMF exposure. Safety was assessed by monitoring the incidence of adverse events during exposure procedures. Adverse events were reported by investigators and their severity was graded using the National Cancer Institute Common Terminology Criteria for Adverse Events (CTCAE). Adverse events were not systematically monitored at other times.

Exploratory patient‐reported quality‐of‐life endpoints included the proportion of patients with improvement and worsening and the time to deterioration in global health status, functional scale, and symptom scale (defined as a ≥10% change in EORTC QLQ–C30 score from baseline). Median overall survival (OS) in the intention‐to‐treat population was an exploratory safety endpoint, and was compared with OS in the historical control cohort.

No tumor assessments or clinical laboratory analyses were performed as part of the study. Routine results from radiological and laboratory assessments of patients were used (with primary physician approval) for ad hoc analyses of objective response rate using Response Evaluation Criteria in Solid Tumors (RECIST) v1.1,^38^ as assessed by investigators and an independent blinded radiologist.

### 2.7 Hemodynamic analyses

Established hemodynamic time series algorithms were used to ensure stability and reproducibility.^27^ We used an auto‐regressive integrated moving‐average model (Autem Mathematical Model)^39^ in part 2 to identify hemodynamic signals of interest in the time series synchronized with the amplitude‐modulation frequencies. These were used as measures of hemodynamic variability induced by EMF exposure. Hemodynamic analysis of data collected in part 2 used power spectral density by fast Fourier transform.^40^

### 2.8 Statistical methods

A minimum sample size of 45 patients with advanced HCC and 45 healthy controls was planned to study hemodynamic regulation. Statistical procedures were non‐parametric: Mann–Whitney U test and/or Kruskal–Wallis analysis of variance (ANOVA) for independent distributions; and Wilcoxon test and/or Friedman ANOVA for related distributions. EORTC QLQ–C30 analysis was performed according to the manual.^37^ Kaplan–Meier analysis and the log‐rank Mantel–Cox test were used to compare OS in the patient cohort and the historical control cohort (two‐sided *α* = 0.05). Stratification factors included Child–Pugh classification, albumin–bilirubin (ALBI) score and line of treatment.

### 2.9 Conduct and oversight

Hospital Sírio‐Libanês was the only study site. All participants gave written informed consent before enrolment. The trial was conducted under the principles of the Declaration of Helsinki and the protocol was reviewed and approved by the institutional review board and ethics committee of Hospital Sírio‐Libanês and by the Comissão Nacional de Ética em Pesquisa. After completion of planned enrolment, the Hospital Sírio‐Libanês ethics committee approved an ongoing compassionate access program following the same protocol with an amended consent form. The trial was registered at ClinicalTrials.gov before enrolment began (NCT01686412).

## 3 RESULTS

### 3.1 Participants

From March 2018 to March 2019, 47 patients with advanced HCC and 51 healthy volunteers were enrolled in parts 1 and 2 of the study (Figure S1). From April 2019 to August 2021 (data cut‐off), an additional 24 patients were enrolled in part 2 under the compassionate access program. Five patients in part 1 were subsequently found to be ineligible and excluded, two with excessive ascites that prohibited decubitus, and three with mixed cholangiocarcinoma on pathology review (Figure S1).

The study population comprised 66 patients and 51 healthy volunteers, who all underwent at least one AutEMdev exposure procedure (Figure 1). Baseline characteristics are presented in Table 1. Median follow‐up was 30 months (95% confidence interval [CI]: 23.9, 36.0) and no participants were lost to follow‐up. Part 1 comprised 26 patients and 32 healthy controls (fixed frequencies) and part 2 comprised 40 patients and 19 healthy volunteers (personalized frequencies). EMF was a monotherapy in 39 patients and a combination therapy in 27; concurrent treatments are shown in Table S1. Three patients in the monotherapy subgroup subsequently received combination therapy following radiological progression.

**TABLE 1:** Baseline demographics and disease characteristics.

	Study population			Historical control cohort (*n* = 45)
	All patients (*N* = 66)	Monotherapy subgroup (*n* = 39)	Combination therapy subgroup (*n* = 27)	
Male, *n* (%)	56 (85)	33 (85)	23 (85)	41 (91)
Age, median (range)	69.5 (32–88)	71 (40–88)	70 (32–85)	66.8 (38–94)
Pathology confirmation, *n* (%)	60 (91)	34 (87)	26 (96)	13 (29)
Extrahepatic metastasis, *n* (%)	31 (47)	16 (41)	14 (52)	20 (44)
Child–Pugh class, *n* (%)				
A	52 (79)	28 (72)	24 (89)	34 (76)
B	14 (21)	11 (28)	3 (11)	11 (24)
ECOG performance status, *n* (%)				
0	56 (85)	31 (79)	25 (93)	NA
1	8 (12)	6 (15)	2 (7)	
2	2 (3)	2 (5)	0 (0)	
BCLC stage, *n* (%)				
B	22 (33)	10 (26)	12 (44)	NA
C	44 (67)	29 (74)	15 (56)	
ALBI grade,^a^ *n* (%)				
1	36 (55)	18 (46)	18 (67)	NA
2	22 (33)	15 (38)	7 (26)	
3	7 (11)	5 (13)	2 (7)	
Previous treatment, *n* (%)				
Chemotherapy	4 (6)	0 (0)	4 (15)	1 (2)
TKI^b^	14 (21)	13 (33)	1 (4)	6 (13)
Immunotherapy	2 (3)	1 (3)	1 (4)	0 (0)
None	46 (70)	25 (64)	21 (78)	36 (80)
Concurrent treatment,^b^ *n* (%)				
Chemotherapy	6 (9)	0 (0)	6 (22)	13 (29)
TKI^c^	27 (41)	[2 (5)]^d^	27 (100)	24 (53)
Immunotherapy^c^	7 (11)	[1 (3)]^d^	7 (26)	2 (4)
None	39 (59)	39 (100)	0 (0)	6 (13)

Abbreviations: ALBI, albumin–bilirubin; BCLC, Barcelona Clinic Liver Cancer; ECOG, Eastern Cooperative Oncology Group; NA, not available; TKI, tyrosine kinase inhibitor.

^a^ Missing in one patient.

^b^ Patients may have received more than one concurrent treatment during the study.

^c^ See Table 2 for details.

^d^ Three patients received standard treatment during follow‐up but were included in the monotherapy group.

### 3.2 Safety

During the 36‐month study period, 488 exposure procedures were performed in patients (median, 7.0 per patient [range, 1–42]) and 51 in healthy volunteers (one per volunteer, per protocol). All healthy volunteers tolerated the procedure. Two excluded patients were unable to tolerate decubitus during the pre‐exposure resting period (which was therefore unrelated to EMF exposure). No serious adverse events were reported during procedures. CTCAE grade 1 somnolence was the only adverse event, occurring in 52/66 patients (78.8%) and in 25/51 healthy volunteers (49.0%). This was characterized by mild but more than usual drowsiness or sleepiness, started within minutes of exposure initiation and ended spontaneously upon completion. All participants were able to resume normal activities after discharge.

### 3.3 Hemodynamics

The hemodynamic analysis included 134.3 h of continuous recording (5 × 10^5^ heartbeats) from the 26 patients in part 1 (11.8 h for healthy volunteers). The technique for identifying hemodynamic signals of interest was applied to 13 patients in part 1 and all 40 patients in part 2. The frequency of variation in hemodynamic signals of interest ranged from 0.9 to 5.2 mHz. Hemodynamic signals of interest occurred more frequently in patients than in healthy volunteers (*η*_*p*_^2^, 0.7973; *p* < 0.0001) and during exposure than non‐exposure in both patients and healthy volunteers (*p* = 0.0039 and *p* < 0.0001, respectively) (Figure 2B,C). Within patients, hemodynamic signals of interest differed significantly across amplitude‐modulation frequencies (*p* = 0.0018). In a detrended fluctuation analysis^41^ the mean value for fluctuations (F[Δn]) was 1.000 (95% CI: 1.006, 1.011), indicating strong correlation of hemodynamic signals of interest with specific amplitude‐modulation frequencies. A power law defined the number of hemodynamic signals of interest occurring in patients as a function of amplitude‐modulation frequency band (Figure 2D,E).^42^

### 3.4 Quality of life

From baseline (before first exposure) to the second, third, and fourth exposure, the majority of patients had improved or stable EORTC QLQ–C30 scores (Table S2). An increasing trend in global health status from baseline was noted over 25 exposures (*p* < 0.0001) (Figure S2). Median time to deterioration was not reached for global health status, 1.28 months (95% CI: 7.14, 15.42) for physical functioning, 11.28 months (4.34, 18.22) for role functioning, and 24.87 months (9.05, 40.69) for functioning score (Figure S2).

### 3.5 Overall survival

During follow‐up, 47/66 patients (71.2%) died from cancer. The median OS of 11.3 months (95% CI: 6.0, 16.6; *N* = 66) was significantly longer than in the in the historical cohort (5.2 months; *p* < 0.0001; *N* = 45; Table 1). Median OS was 16.0 months (95% CI: 4.4, 27.6) for combination therapy and 12.0 months (6.4, 17.6) for monotherapy. A subgroup analysis of OS is presented in Table 2. The median duration of sorafenib treatment was 3.4 months (95% CI: 0.0, 9.0) before enrolment (*n* = 10) and 6.7 months (0.0, 13.8) during study combination therapy (*n* = 18).

**TABLE 2:** Subgroup analysis of OS.

	Patients, *n*	Deaths, *n*	Median OS, months (95% CI)
All patients	66	47	11.3 (6.0, 16.6)
Part 1	26	21	11.4 (5.6, 17.0)
Part 2	40	26	10.6 (0.0, 21.4)
EMF combination therapy	27	18	16.0 (4.4, 27.6)
EMF monotherapy	39	29	12.0 (6.4, 17.6)
First‐line EMF	46	29	18.4 (11.9, 24.9)
Second‐line EMF	15	13	5.2 (0.0, 12.8)
ALBI grade 1	36	21	18.9 (13.2, 24.6)
ALBI grade 2	22	18	5.2 (0.0, 10.4)
First‐line EMF combination therapy	23	15	18.4 (12.1, 24.7)
First‐line EMF monotherapy	23	14	14.6 (1.2, 28.1)
First‐line EMF and ALBI grade 1	25	16	21.6 (1.4, 41.8)
First‐line EMF and ALBI grade 2	14	8	19.3 (4.3, 34.2)

Abbreviations: ALBI, albumin–bilirubin; CI, confidence interval; EMF, electromagnetic field; OS, overall survival.

### 3.6 Radiological responses

**FIGURE 3:**
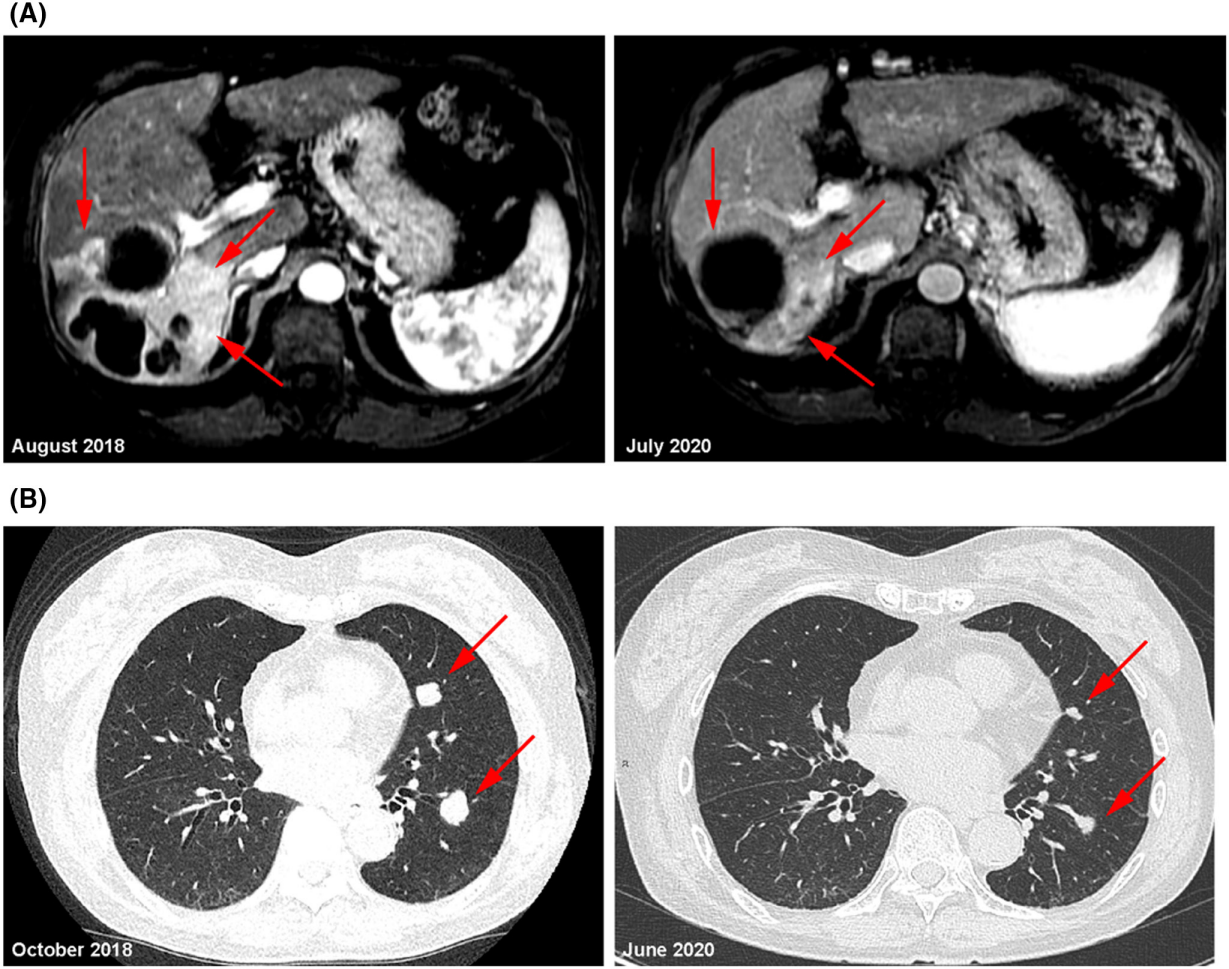
Imaging of two patients with partial radiographic responses. (A) Metastatic liver disease in a patient receiving 35 AutEMdev EMF exposure procedures as monotherapy (magnetic resonance imaging). (B) Metastatic lung disease in a patient receiving 31 AutEMdev EMF exposure procedures in combination with first‐line lenvatinib (computed tomography). Red arrows indicate tumors. Abbreviations: AutEMdev indicates Autem electromagnetic device; EMF, electromagnetic field.

By independent review in 37/66 patients (56.1%) with evaluable scans, the objective response rate by RECIST 1.1 was 6/19 (32%) for combination therapy and 3/18 (17%) for monotherapy, with complete responses in 1/19 (5%) and 2/18 (11%) patients, respectively (one with Child–Pugh class B) (Table 3). By investigator assessment in 47/66 patients (71.2%) with evaluable scans, the objective response rate was 10/47 (21.3%) (Table 3). Images from two patients with durable partial responses are shown in Figure 3.

**TABLE 3:** RECIST v1.1 responses among evaluable patients.

	Independent review			Investigator read (*n* = 47)^a^
	Monotherapy (*n* = 18)	Combination therapy (*n* = 19)	Overall (*n* = 37)^a^	
Complete response, *n* (%)	3 (17)^b^	1 (5)	4 (11)^b^	4 (9)^b^
Partial response, *n* (%)	1 (6)	5 (26)	6 (16)	7 (15)
Stable disease, *n* (%)	9 (50)	10 (53)	19 (51)	25 (53)
Progressive disease, *n* (%)	5 (28)	3 (16)	8 (22)	11 (23)
Disease control rate, %	72^c^	84	76^c^	74^c^
Objective response rate, %	17	32	24	21

Abbreviations: AutEMdev, generator of electromagnetics, EMF, electromagnetic field; RECIST, Response Evaluation Criteria in Solid Tumors.

^a^ Not all of the 66 patients in the study population had follow‐up scans available. 10 patients with data were not evaluable by independent review.

^b^ One of these four patients had a complete resection after EMF monotherapy with the AutEMdev and remained cancer‐free.

^c^ Excluding patient who received a complete resection and remained cancer‐free.

## 4 DISCUSSION

This feasibility trial confirmed the safety of EMF therapy with the AutEMdev in patients with advanced HCC, regardless of liver function (Child–Pugh class A–B), combination with systemic treatments and previous lines of therapy. No serious adverse events were reported in patients or healthy volunteers during any EMF exposure procedure. Quality of life was maintained among patients undergoing repeated exposures every 2–4 weeks. The extended OS compared with a historical control cohort indicated that AutEMdev EMF exposure was not harmful, and may suggest potential efficacy when combined with the radiological responses observed in a minority of patients.

AutEMdev EMF exposure caused subtle but reproducible alterations in hemodynamic regulation, as assessed using beat‐to‐beat heart rate variability measurements.^27^ Similar findings have been reported, although the underlying physiopathology remains unclear.^28^, ^29^, ^30^, ^43^ Furthermore, hemodynamic variability induced by EMF exposure appeared to be more common in patients with cancer than controls, potentially because of alterations in homeostasis induced by the tumor load, although this requires further investigation. Specific amplitude‐modulation frequencies correlated with hemodynamic signals of interest and obeyed a power law, indicating a non‐random relationship. The significance of this finding remains unclear, and the present study did not aim to investigate whether the relationship between hemodynamic variability and specific amplitude‐modulation frequencies is associated with clinical efficacy or safety signals. Further studies are required to test the hypothesis that personalized amplitude‐modulation frequencies are associated with both hemodynamic signals of interest and potential clinical benefits.

Mild, self‐limiting somnolence was a common adverse event in individuals receiving AutEMdev EMF, consistent with previous findings.^44^, ^45^, ^46^ In contrast, in patients receiving TTFields, dermatological adverse events are common and skin toxicity may limit exposure.^47^ EMF exposure can affect α waves and is an option for treatment of depression and pain.^10^ We speculate that an effect of EMF on excitable cells may underlie both hemodynamic alterations and somnolence.^10^ Further studies are required to investigate this possibility.

OS was similar in patients receiving AutEMdev EMF monotherapy and combination therapy, including first‐line EMF. A trend toward increased duration of sorafenib treatment was observed among patients receiving combination therapy compared with pre‐enrolment. Furthermore, complete and partial RECIST v1.1 responses were observed in patients receiving EMF as a monotherapy and in combination with tyrosine kinase inhibitors. These exploratory findings warrant future investigation of the AutEMdev using personalized treatment frequencies in a prospective randomized‐controlled efficacy study.

Limitations of this study include the open‐label, single‐site design, and lack of a sham exposure procedure. This spared patients from placebo interventions and ensured consistency. Safety was only monitored during procedures, and did not include liver function tests. Few patients received PD(L)‐1 inhibitors because these were not approved in Brazil when the study began. Sources of significant potential bias included: wide eligibility criteria, selection of patients willing and able to participate; patients' freedom to repeat exposures; systemic treatment flexibility; and heterogeneity in the historical control arm. Another limitation was that the study did not aim to investigate the potential relationship between hemodynamic signals of interest and clinical efficacy or somnolence side‐effects. Strengths of the study include the blinded independent radiographic review, the evaluation of OS and the use of a validated patient‐reported outcome.

In conclusion, EMF treatment with the AutEMdev had an excellent safety profile in patients with advanced HCC, as both monotherapy and combination therapy. There was evidence for maintained quality of life among patients who remained on treatment. Hemodynamic variability during exposure may provide a biological surrogate for identification of optimal amplitude‐modulation frequencies in each patient. Whether these personalized frequencies are also optimal for potential anti‐tumor effects remains to be established. This study was not designed to assess efficacy, but the positive exploratory trends warrant further investigation.

## AUTHOR CONTRIBUTIONS

**Fernanda Capareli:** Investigation (lead). **Frederico Costa:** Investigation (equal) and Methodology (equal). **Jack Tuszynski:** Methodology (equal). **Micelange Carvalho de Sousa:** Investigation (equal). **Yone de Camargo Setogute:** Investigation (equal). **Pablo Diego Santana:** Investigation (equal). **Luciana Carvalho:** Investigation (equal). **Elizabeth Santana dos Santos:** Investigation (equal). **Brenda Gumz:** Investigation (equal). **Jorge Sabbaga:** Investigation (equal). **Tiago Bianc de Castria:** Investigation (equal). **Denis Leonardo Jardim:** Investigation (equal). **Daniela de Freitas:** Investigation (equal). **Natally Horvat:** Data curation (equal). **Regis França Otaviano:** Data curation (equal). **Leonardo Testagrossa:** Data curation (equal). **Tiago Costa:** Data curation (equal). **Tatiana Zanesco:** Investigation (equal). **Antonio Francisco Iemma:** Data curation (equal). **Ghassan K Abou‐Alfa:** Writing – review and editing (lead).

## FUNDING INFORMATION

Hospital Sírio‐Libanês sponsored this trial and provided the non‐invasive hemodynamic device. The study was part‐funded by National Institutes of Health/National Cancer Institute Cancer Center Support Grant P30 CA008748 to Natally Horvat. The AutEMdev was provided by Autem Therapeutics under a research development agreement with Hospital Sírio‐Libanês. Medical writing support was provided by Oxford PharmaGenesis, Oxford, UK, funded by Autem Therapeutics. Clinical records were reviewed by an independent auditor from Emergo (Austin, TX, USA) and radiological review was conducted by an independent blinded radiologist from Charité–Universitätsmedizin Berlin (Berlin, Germany), funded by Autem Therapeutics.

## CONFLICT OF INTEREST STATEMENT

TBC: Honoraria, consultancy fees or research funding from AstraZeneca, Bayer, Eli Lilly, Genentech/Roche, Bristol Myers Squibb, Merck and Merck Sharp & Dohme. FC, AFI, and JA: Stock in and honoraria from Autem Therapeutics and patents relating to the AutEMdev. YdCS: Honoraria from Bayer, Merck and Novartis and independent contractor at Hospital Sirio‐Libanês. GKA‐A: Institutional research support from Arcus, Astra Zeneca, BioNtech, BMS, Celgene, Flatiron, Genentech/Roche, Genoscience, Incyte, Polaris, Puma, QED, Silenseed, Yiviva; consulting fees from Adicet, Alnylam, Astra Zeneca, Autem, Beigene, Berry Genomics, Boehringer Ingelheim, Celgene, Cend, CytomX, Eisai, Eli Lilly, Exelixis, Flatiron, Genentech/Roche, Genoscience, Helio, Helsinn, Incyte, Ipsen, Merck, Nerviano, Newbridge, Novartis, QED, Redhill, Rafael, Servier, Silenseed, Sobi, Vector, Yiviva; registered patent: PCT/US2014/031545 filed on March 24, 2014, and priority application Serial No.: 61/804,907; Filed: March 25, 2013. DLJ honoraria, consultancy fees or research funding from AstraZeneca, Bayer, Janssen, Astellas, Genentech/Roche, Bristol Myers Squibb, Merck and Merck Sharp & Dohme.

## Supporting information


Data S1.

## Data Availability

Data sharing is not applicable to this article as no new data were created or analyzed in this study.
