# Supplementary material for: Low‐energy amplitude‐modulated electromagnetic field exposure: Feasibility study in patients with hepatocellular carcinoma
Source: Cancer Med. 2023 May 15;12(11):12402–12. doi: 10.1002/cam4.5944 (PMC10278519; doi:10.1002/cam4.5944)
Supplement: Supplementary file 1 — Data S1. [file CAM4-12-12402-s001.docx]

# Supplementary Information

## Supplementary Tables

#### Supplementary Table 1. Concurrent treatment in the combination therapy subgroup by line of therapy.

|  | **Tyrosine kinase inhibitor (*n* = 27)** | | |  | **Immunotherapy (*n* = 7)** | |
| --- | --- | --- | --- | --- | --- | --- |
|  | **Sorafenib** | **Lenvatinib** | **Regorafenib** |  | **Atezolizumab plus bevacizumab** | **Nivolumab** |
| First-line, *n* | 15 | 5 | 0 |  | 1 | 1 |
| Second-line, *n* | 3 | 0 | 4 |  | 4 | 1 |

#### Supplementary Table 2. Categorical analysis of change in EORTC QLQ–C30 scores from baseline.

|  | **Proportion of patients (*N* = 66)** | | |
| --- | --- | --- | --- |
|  | **Second exposure** | **Third exposure** | **Fourth exposure** |
| **Symptom scales, %** | |  |  |
| Improved | 17 | 26 | 30 |
| Stable | 72 | 58 | 57 |
| Worsened | 11 | 16 | 14 |
| **Global health status, %** | |  |  |
| Improved | 26 | 30 | 30 |
| Stable | 49 | 42 | 43 |
| Worsened | 25 | 28 | 27 |
| **Physical functioning, %** | |  |  |
| Improved | 21 | 26 | 19 |
| Stable | 58 | 47 | 59 |
| Worsened | 21 | 28 | 22 |
| **Role functioning, %** | |  |  |
| Improved | 30 | 35 | 32 |
| Stable | 49 | 42 | 41 |
| Worsened | 21 | 23 | 27 |
| **Functional scales, %** | |  |  |
| Improved | 23 | 30 | 32 |
| Stable | 58 | 49 | 46 |
| Worsened | 19 | 21 | 22 |

Improvement was defined as an increase of ≥10% from baseline (before first exposure) to before subsequent exposures; worsening was defined as a decrease of ≥10%.

*EORTC QLQ-30* European Organisation for Research and Treatment of Cancer Quality of Life Questionnaire Core 30.

## Supplementary Figures

#### Supplementary Fig. 1 CONSORT diagram showing patient disposition.


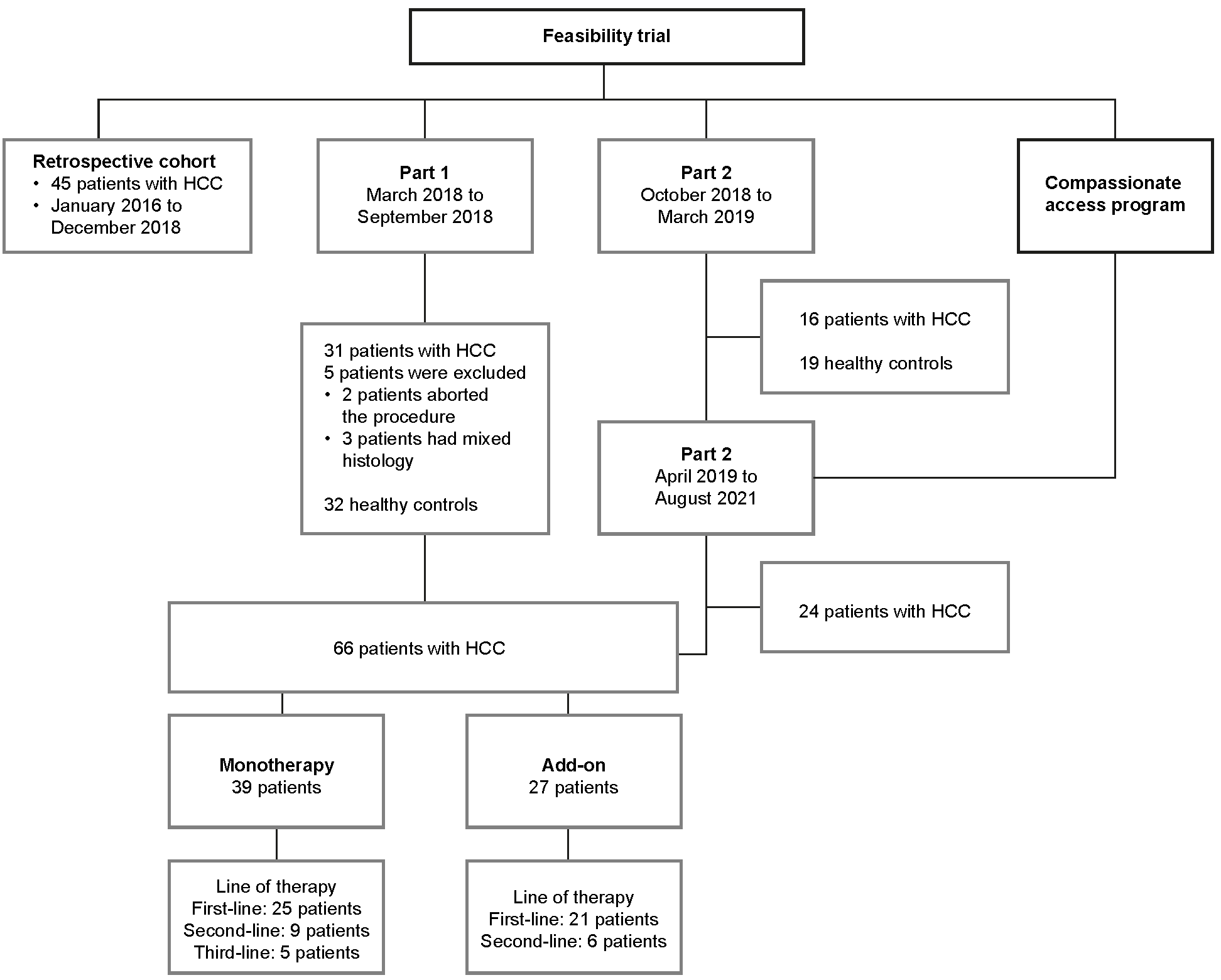


*CONSORT* Consolidated Standards of Reporting Trials, *HCC* hepatocellular carcinoma.

#### Supplementary Fig. 2 Health-related QoL. a time to deterioration in EORTC QLQ–C30 for global health, physical functioning, role functioning and functioning score. b mean EORTC QLQ–C30 global health scores for each exposure procedure from all patients. c mean EORTC QLQ–C30 symptom scores for each exposure procedure from all patients. Numbers in brackets in b and c indicate number of patients with data.


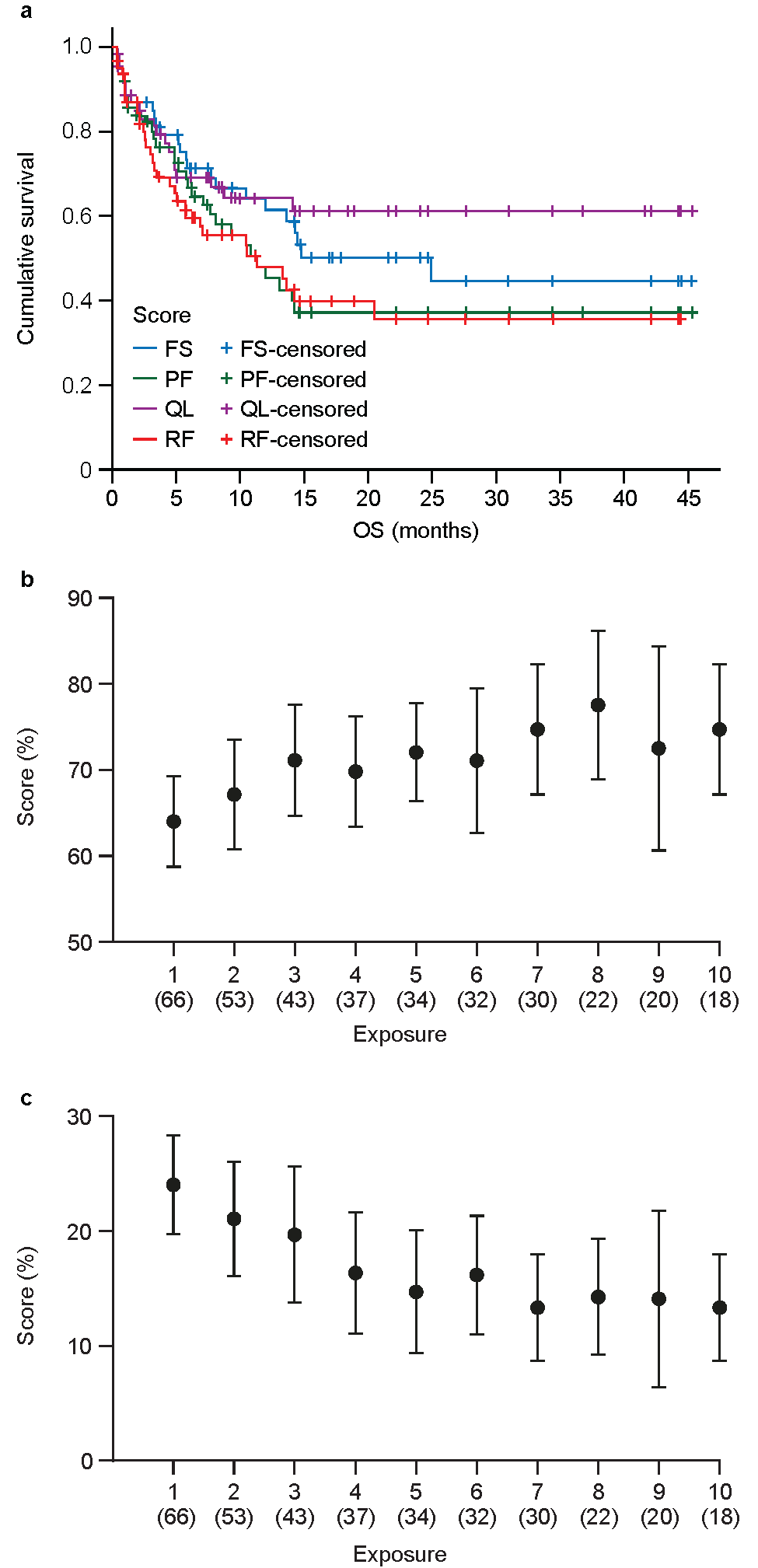


*EORTC QLQ-30* European Organisation for Research and Treatment of Cancer Quality of Life Questionnaire Core 30, *FS* functioning score, *OS* overall survival, *PF* physical functioning, *QL* global health, *QoL* quality of life, *RF* role functioning.
